# Differences in Grey Matter Concentrations and Functional Connectivity between Young Carriers and Non-Carriers of the APOE ε4 Genotype

**DOI:** 10.3390/jcm13175228

**Published:** 2024-09-03

**Authors:** Carlos Muñoz-Neira, Jianmin Zeng, Ludmila Kucikova, Weijie Huang, Xiong Xiong, Graciela Muniz-Terrera, Craig Ritchie, John T. O’Brien, Li Su

**Affiliations:** 1 Artificial Intelligence & Computational Neuroscience Group (AICN Group), Sheffield Institute for Translational Neuroscience (SITraN), Division of Neuroscience, School of Medicine and Population Health, Faculty of Health, University of Sheffield, Sheffield S10 2HQ, UK; carlos.munoz@sheffield.ac.uk (C.M.-N.); lkucikova1@sheffield.ac.uk (L.K.); weijiehuang@mail.bnu.edu.cn (W.H.); xx839@cam.ac.uk (X.X.); 2 Old Age Psychiatry Research Group (OAP Group), Department of Psychiatry, School of Clinical Medicine, University of Cambridge, Cambridge CB2 0SZ, UK; john.obrien@medschl.cam.ac.uk; 3 Sino-Britain Centre for Cognition and Ageing Research, Faculty of Psychology, Southwest University, Chongqing 400715, China; 4 Insigneo Institute for In Silico Medicine, University of Sheffield, Sheffield S1 3JD, UK; 5 School of Systems Science, Beijing Normal University, Beijing 100875, China; 6 School of Information and Communication Engineering, Beijing University of Posts and Telecommunications, Beijing 100876, China; 7 Edinburgh Dementia Prevention, Centre for Clinical Brain Sciences, University of Edinburgh, Edinburgh EH4 2XU, UK; g.muniz@ed.ac.uk (G.M.-T.); craig.ritchie@ed.ac.uk (C.R.); 8 Ohio University Heritage College of Osteopathic Medicine, Ohio University, Athens, OH 45701, USA; 9 Scottish Brain Sciences, Edinburgh EH12 9DQ, UK

**Keywords:** young adults, APOE ε4, structural brain imaging, functional brain imaging, voxel-based morphometry, seed-based connectivity, magnetic resonance imaging, resting state functional magnetic resonance imaging, Alzheimer’s disease

## Abstract

**Background**: The pathophysiology of Alzheimer’s disease (AD) may begin developing years or even decades prior to the manifestation of its first symptoms. The APOE ε4 genotype is a prominent genetic risk for AD that has been found to be associated with brain changes across the lifespan since early adulthood. Thus, studying brain changes that may occur in young adults with an APOE ε4 status is highly relevant. **Objective**: Examine potential differences in grey matter (GM) and functional connectivity (FC) in brains of cognitively healthy young APOE ε4 carriers and non-carriers, denoted here as ε4(+) and ε4(−), respectively. **Methods**: Three Tesla magnetic resonance imaging (MRI) brain scans were acquired from cognitively healthy young participants aged approximately 20 years (n = 151). Voxel-based morphometry (VBM) analysis was employed to identify potential structural differences in GM between ε4(+) and ε4(−). In a subsequent seed-based connectivity (SBC) analysis, brain regions that structurally differed in the VBM analysis were considered as seeds and correlated with all the remaining voxels across the brains to then measure the differences in FC between groups. **Results**: The VBM analysis suggested that ε4(+) (n = 28) had greater GM densities relative to ε4(−) (n = 123) in the left hippocampus and the left posterior insula (p_uncorr_ < 0.001). However, the effect did not survive the correction for multiple comparisons, suggesting minimal structural differences in this age range. In contrast, the SBC analysis indicated that ε4(+) exhibited significantly decreased FC between the left hippocampus and areas of the left middle temporal gyrus (n = 27) compared to ε4(−) (n = 102). These results remained significant after multiple comparisons (p_FDR_ < 0.05). Lastly, no statistically significant differences in FC between groups were observed for the left insular seed (p_FDR_ > 0.05). **Discussion**: These results suggest early structural and functional brain changes associated with the APOE ε4 genotype on young adults. Yet, they must be cautiously interpreted and contrasted with both older adults with genetic risk for AD and patients diagnosed with AD.

## 1. Introduction

Dementia is a global challenge due to its high prevalence in older people [1,2] and its associated high costs in health and social care [3]. Alzheimer’s disease (AD) is the most common form of dementia [4], contributing to more than half of all dementia cases in those aged above 65 years [5]. Whilst AD symptoms can be typically observable in later life [6,7], AD pathophysiology may begin developing years or even decades earlier [8,9]. Hence, focusing research on young individuals with genetic risks to develop AD can shed light on its pathological underpinnings and clinical markers across the lifespan. Furthermore, this approach may enhance a timely diagnosis, facilitate early clinical and lifestyle interventions, and prompt prevention efforts [10,11].

APOE ε4 is the most prominent genetic risk factor for AD [12]. Estimates suggest that it is responsible for approximately 20% of all the cases of dementia and between 65% and 75% of all the cases of sporadic AD [13]. By contrast, other APOE alleles like ε2 or ε3 may have a neuroprotective effect [14] or a rather neutral/defensive influence [15], respectively. Likewise, brain imaging studies that conduct comparisons between APOE ε4 carriers and non-carriers, denoted as ε4(+) and ε4(−), respectively, in this paper, may depict premature structural and/or functional brain changes that could be taken later as early biomarkers for AD.

For example, structural neuroimaging through magnetic resonance imaging (MRI) has revealed grey matter (GM) differences between ε4(+) and ε4(−) of different ages over the course of life [16]. Among young adults (aged ~20 years), the right hippocampus has shown significantly smaller sizes in ε4(+) compared to ε4(−) [17]. Nevertheless, other studies found no significant hippocampal differences at this age [18]. In addition, cognitively healthy middle-aged adults (aged 40–59 years) ε4(+) exhibited significantly reduced volumes of the molecular layer of the hippocampi compared to ε4(−), although the entire volume of these areas may be unchanged [19]. Similarly, ε4(+) individuals in midlife have presented reduced cortical thickness over entorhinal and subiculum areas [20]. Altogether, such findings seem to be consistent with the patterns of neurodegeneration observed in typical AD patients, especially in the hippocampi [21]. 

In addition to structural changes, previous studies conducted with resting state functional MRI (rsfMRI) have indicated variable alterations in functional connectivity (FC) caused by APOE ε4 in different age groups. For instance, cognitively intact young ε4(+) in their 20s have shown increased FC in a brain network connecting hippocampal with sensorimotor areas [22]. In contrast, when taking a region of interest (ROI) like the hippocampus or the cuneus bilaterally, cognitively healthy middle-aged ε4(+) (up to 62 years old) can show a decreased FC towards adjacent structures commonly affected in AD [23,24]. Additionally, the default mode network (DMN), which spans throughout the hippocampal, cingulate, and temporoparietal regions [25], appears to be shaped by the APOE ε4 status in different forms [26,27]. In comparison with ε4(−), healthy young adults ε4(+) can exhibit an increased [28] or decreased FC [29], whereas healthy middle-aged adults ε4(+) may present an unaffected [30] or decreased [24] FC across this brain circuitry.

In sum, prior research has suggested heterogeneous effects of the APOE ε4 allele on brain changes over different ages. Thus, it remains unclear how APOE ε4 interacts with both brain structure and function at the same time in early life. A data-informed methodology may be valuable to address this issue due to its ability to capture nuanced variations and complexities within the brain structure and functioning within a given sample. In the current study, we combined both structural and functional neuroimaging to examine potential brain differences in GM and FC in brains of cognitively healthy young ε4(+) and ε4(−), adopting a data-driven multimodal MRI approach. We consider that their concatenation may provide an exhaustive revision of the impact of the APOE ε4 genotype on brain integrity.

## 2. Materials and Methods

### 2.1. Participants, Study Design, and Ethics

A convenience sample of 151 cognitively healthy young adults aged between 17 and 22 years was recruited from Southwest University, Chongqing, China. The participants were divided into ε4(+) and ε4(−) groups, according to their APOE genotype. Participants underwent genetic testing and 3 Tesla (T) MRI brain scans, including T1 and rsfMRI sequences. Age, years of education, and sex were recorded to report a demographic characterization of the sample. Demographics, as well as structural and functional brain images, were analyzed as detailed later. All participants provided written informed consent. This study was approved by the Ethic Committee of Psychological Research at Southwest University, Chongqing, China. Additionally, ethical approval from the University of Sheffield was obtained through an internal self-declaration form (Reference Number 043330; approval date 6 September 2021).

### 2.2. Data Collection

#### 2.2.1. Genotyping

The APOE ε4 allele status was evaluated through a salivary sample utilizing the MassARRAY system (Agena iPLEX assay, San Diego, CA, USA), wherein the specimens were tested against a multiplex PCR reaction, followed by mass spectrometry, to identify specific alleles and then code the respective specific genotypes.

#### 2.2.2. Brain Imaging

MRI brain scans were acquired from all the subjects at Southwest University in Chongqing, China, employing a high-resolution 3 T Siemens TrioTim MRI scanner, which captured both structural and functional sequences. Three-dimensional T1-weighted magnetization-prepared rapid gradient-echo (MPRAGE) images were taken according to the following parameters: 160 slices of 1 mm of thickness, repetition time (TR) = 2.3 s, echo time (TE) = 0.00298 s, inversion time (TI) = 0.9 s, flip angle = 9°, Field of View (FOV) = 240 × 256 mm^2^, voxel size = 1 × 1 × 1 mm^3^, GRAPPA factor 2, and total acquisition time = ~15–20 min. Resting-state echo planar images were obtained from participants whilst they were at rest and were asked to maintain their eyes shut, refraining themselves from thinking about anything specific. These sequences were taken then considering the following parameters: 35 slices of 3mm of thickness, TR/TE = 2 s/0.03 s, flip angle = 80°, FOV = 192 × 192 mm^2^, voxel size = 3 × 3 × 3 mm^3^, slice acquisition = interleaved with total 330 measurements, and total acquisition time = ~10–15 min.

### 2.3. Data Analysis

#### 2.3.1. Demographic Data

All statistical analyses were carried out at a level of significance (*p*-value) lower than 0.05 (*p* < 0.05; two-tailed) with the software Jasper’s Amazing Statistical Programme Version 0.18.3 for Windows downloaded from https://jasp-stats.org/ (accessed on 6 September 2021) [31]. Age and years of education were tested for normality through Shapiro–Wilk tests to decide on the utilization of parametric or non-parametric statistics for the respective comparisons made between ε4(+) and ε4(−), either *t*-tests for the former or Mann–Whitney *U* tests for the latter. Comparisons between groups regarding sex were carried out with chi-square (X^2^) tests.

#### 2.3.2. Structural Brain Imaging Analysis

Structural neuroimaging analysis was undertaken through voxel-based morphometry (VBM) employing the software Statistical Parametric Mapping 12 (SPM12) [32] to identify differences in GM concentrations [33] between ε4(+) and ε4(−). We firstly segmented the brain tissue into GM, white matter (WM), and cerebrospinal fluid (CSF) using the Computational Anatomy Toolbox 12 (CAT12) [34,35]. Images were normalized to the Montreal Neurological Institute (MNI) space, as well as smoothed with an 8 mm full width at half-maximum (FWHM) filter considering a mask with an absolute threshold of 0.05 [33].

A two-sample *t*-test model was used to compare structural brain differences between ε4(+) and ε4(−). The total intracranial volume (TIV), age, years of education, and sex were entered into this model as covariates. Identification and labeling of statistically significant brain areas was done with the Neuromorphometrics brain atlas incorporated in SPM12. Significant results for this analysis considered family-wise error-corrected *p*-values smaller than 0.05 (p_FWEcorr_ < 0.05).

#### 2.3.3. Functional Brain Imaging Analysis

Functional neuroimaging analysis was performed through seed-based connectivity (SBC) analysis using the software CONN (RRID:SCR_009550) [36] to explore the presence of differences in FC between ε4(+) and ε4(−). Pre-processing of the rsfMRI sequences was conducted with a default pipeline that involved functional realignment and unwarp (co-registration to a reference image using a least squares approach and a 6 parameter transformation and resampling with b-spline interpolation); slice-timing correction (images organized in interleaved Siemens order resampling misaligned BOLD timeseries to a common mid-acquisition time); outlier identification (an artifact detection tool captured acquisitions with framewise displacement above 0.9 mm or global Blood Oxygenation Level Dependent -BOLD- signal changes above 5 standard deviations); direct segmentation of T1 MRI (into GM, WM, and CSF); normalization (into the standard MNI space); and functional smoothing (spatial convolution with a Gaussian kernel of 8 mm FWHM) [37,38].

Denoising continued following a standard pipeline to refine the functional data incorporating regressions of possible confounding variables, namely WM and CSF (10 and 5 CompCor noise components, respectively), motion parameters and their first-order derivatives, outlier scans, session and task effects with their first-order derivatives and linear trends (12, 48, 8, and 2 factors, accordingly) for each functional run. Then, a bandpass frequency filtering of the BOLD timeseries was implemented, setting a range between 0.008 and 0.09 Hz, to also consider low-frequency fluctuations of other signals [37,38].

Pre-processing and denoising were followed by the SBC analysis, which permits to predefine seeds to examine their connectivity with the rest of the brain. In the present study, brain areas where GM concentrations differed significantly in the previous VBM analysis were taken as seeds. The FC values in these seeds were then correlated with FC values of all the remaining voxels across participants’ brains to measure differences between ε4(+) and ε4(−). It should be noted that the FC for the chosen seeds was explored considering the predetermined regions from the Oxford-Harvard atlas offered by CONN, which almost fully matched the brain areas derived from the VBM analysis.

The SBC analysis required the completion of 2 consecutive stages. At the first-level analysis, SBC maps were estimated by computing pairwise Fisher-transformed bivariate correlation coefficients between the voxels of the seeds under study and the other voxels belonging to the predefined areas that CONN provides across the entire brain. At the second-level (group-level) analysis, FC was then compared between ε4(+) and ε4(−) through the configuration of general linear models adjusted for TIV, age, years of education, and sex as covariates. The SBC maps generated were corrected for multiple comparisons employing False Discovery Rate (FDR)-corrected *p*-values smaller than 0.05 (p_FDR_ < 0.05). The outcomes with the final group differences obtained from such maps were converted to *t*-statistics and overlaid onto the standard MNI template for visualization purposes [37,38].

## 3. Results

### 3.1. Demographic Characterization of the Sample

Table 1 displays a summary of the demographic characteristics of the cohort. It should be noticed that some rsfMRI images did not pass quality control for motion and other artefacts, so these participants were excluded from the FC analysis. Distributions for age and years of education were statistically different from normal (Shapiro–Wilk tests of 0.97, *p* = 0.003, and 0.79, *p* < 0.001, respectively). The ε4(+) and ε4(−) groups were not different in terms of age (Mann–Whitney *U* = 1919.50, *p* = 0.35 for structural brain imaging analysis and Mann–Whitney *U* = 1497.50, *p* = 0.49 for functional brain imaging analysis), years of education (Mann–Whitney *U* = 2020.50, *p* = 0.15 for structural brain imaging analysis and Mann–Whitney *U* = 1619.50, *p* = 0.16 for functional brain imaging analysis), and sex (chi-square (X^2^) = 0.01, *p* = 0.92 for structural brain imaging analysis and X^2^ = 0.34, *p* = 0.56 for functional brain imaging analysis).

### 3.2. Structural Brain Differences between ε4(+) and ε4(−)

**Figure 1:**
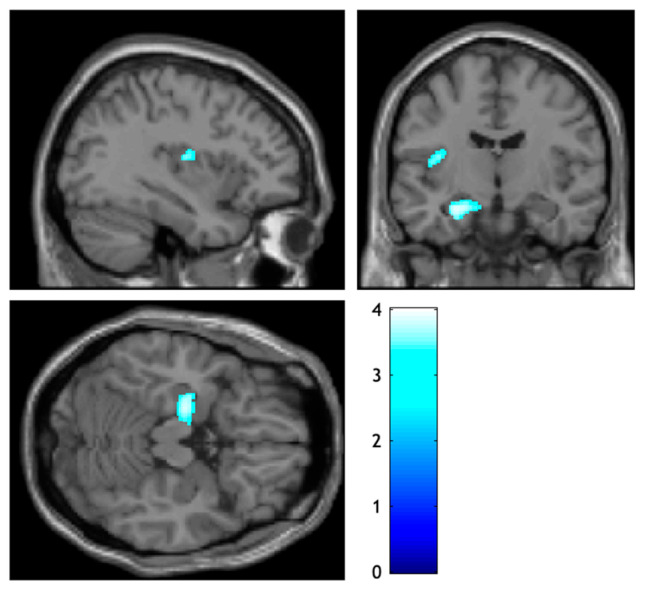
Structural differences between APOE ε4 carrier and APOE ε4 non-carrier groups. ε4(+) exhibited greater volume densities in the hippocampus and the posterior insula across the left hemisphere compared to ε4(−) (p_uncorr_ < 0.001 at the peak level). Uncorrected results mapped onto a single subject template taken from SPM12. ε4(+) = APOE ε4 carriers; ε4(−) = APOE ε4 non-carriers.

According to the VBM analysis conducted, no statistically significant differences in GM concentrations were observed between ε4(+) and ε4(−) after correction for multiple comparisons (p_FWEcorr_ > 0.05). However, when considering a less stringent criterion, an uncorrected GM difference between groups was found. ε4(+) exhibited greater GM densities relative to ε4(−) in the left hippocampus and the left posterior insula with a peak-level threshold (p_uncorr_ < 0.001) (Table 2 and Figure 1).

### 3.3. Functional Brain Differences between ε4(+) and ε4(−)

**Figure 2:**
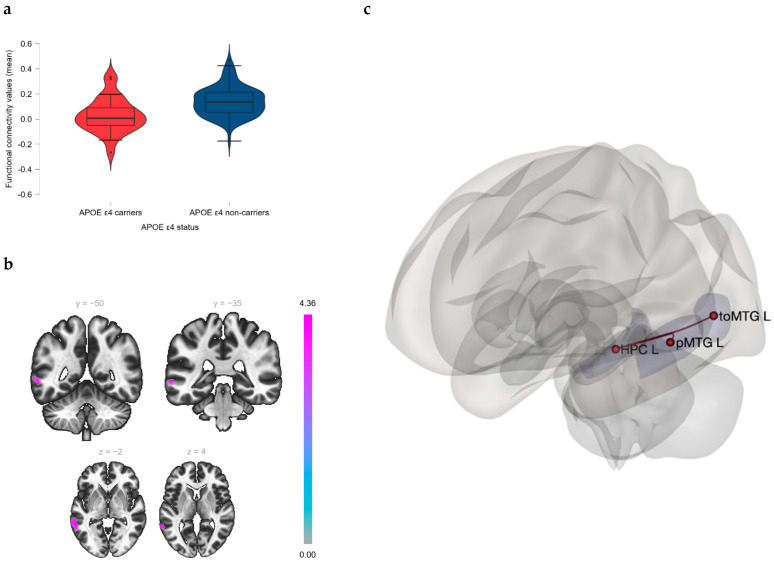
Differences in FC between ε4(+) and ε4(−): Seed-based connectivity analysis. (**a**) Violin plots indicating a significant reduction of FC (p_FDR_ < 0.05) between HPC L and both pMTG L and toMTG L in ε4(+) compared to ε4(−). (**b**) Brain areas indicating statistically significant group differences (effect of the APOE ε4 genotype, p_FDR_ < 0.05) in the hippocampal FC between ε4(+) and ε4(−), according to the SBC analysis. Taking the HPC L as the seed, ε4(+) showed a statistically significant reduction in FC with both pMTG L and toMTG L in comparison with ε4(−) (p_FDR_ < 0.05). (**c**) A three-dimensional model of a brain representing the pattern of reduced FC described previously. FC = functional connectivity; p_FDR_ = False Discovery Rate *p*-value; SBC = seed-based connectivity; HPC L = left hippocampus; pMTG L = left posterior region of the middle temporal gyrus; toMTG L = left temporooccipital division of the left middle temporal gyrus; ε4(+) = APOE ε4 carriers; ε4(−) = APOE ε4 non-carriers.

The subsequent SBC analysis took both the hippocampus and the posterior insula from the left hemisphere as seeds based on the uncorrected clusters from the previous VBM analysis. Statistically significant differences corrected for multiple comparisons were found in FC between the groups according to this SBC analysis (p_FDR_ < 0.05). In contrast with ε4(−), ε4(+) showed a significantly decreased FC between the left hippocampus and the posterior region of the left middle temporal gyrus (MTG), as well as between the left hippocampus and the temporooccipital division of the left MTG (p_FDR_ < 0.05) (Table 3 and Figure 2a,b). An SBC map affected for ε4(+) in these brain areas can therefore be delineated (Figure 2c). No significant results were observed in terms of FC between the left posterior insula and voxels belonging to any other particular area across the brain (p_FDR_ > 0.05) (Table 3).

## 4. Discussion

Our results from the VBM analysis showed no statistically significant differences in GM density between young ε4(+) and ε4(−) after correction for multiple comparisons. Nevertheless, an uncorrected difference was observed in which ε4(+) exhibited greater concentrations of GM across the left hippocampus and the left posterior insula compared to ε4(−). Using the clusters from the VBM analysis as seeds, our findings from the subsequent SBC analysis revealed that, after the correction of multiple comparisons, ε4(+) presented a statistically significantly reduced FC between the left hippocampus (considered as a seed) and posterior and temporooccipital zones of the left MTG. Differently, non-significant differences between groups in terms of FC were observed for the left posterior insula.

The structural VBM analysis showed an uncorrected increased left hippocampal volume in young ε4(+) relative to ε4(−) that is consistent with the mixed findings at this age range discussed in the Introduction section. In comparison with ε4(−), ε4(+) also around the age of 20 years old have shown increased volumes of GM in the right entorhinal cortex [39], which is a well-known region highly associated with the hippocampus structurally and functionally. At the same time, the hippocampi’s volumes seem to increase from early development until roughly this age [40]. Conversely, an absence of differences in the GM volumes of the hippocampus [18] and the MTL entirely [41] have also been observed between young ε4(+) and ε4(−) at this younger age. Opposite to our results, other research has found that young carriers of APOE ε4 and ε3 alleles have presented similar hippocampal sizes, although significantly reduced relative to those from carriers of the APOE ε2 allele [42].

At later stages of life, ε4(+) aged between 20 and 40 have presented significantly smaller sizes in their hippocampi compared to ε4(−) [17]. Correspondingly, a reduction in hippocampal volumes progresses throughout the middle age and becomes more noticeable in older age [40]. This is in line with the significantly decreased size of the hippocampal molecular layer encountered in middle-aged adult carriers of this genotype [19], the significant reduction of certain hippocampal subfields seen in adults with a family history of dementia in their midlife [43], and the well-documented hippocampal and MTL atrophy that occurs in AD [44,45]. Moreover, individuals susceptible to being diagnosed with AD in the short, middle, or long term due to familial or genetic factors, especially ε4(+), have exhibited involvement of areas near the hippocampi such as GM loss across the MTL and precuneus, along with reductions of WM density in the fornix, cingulum, and corpus callosum [46]. Altogether, these findings may suggest that the APOE ε4 genotype could play diverse roles at different stages across the human lifespan.

In the subsequent FC analysis, it was considered that selecting a seed as a ROI from uncorrected VBM results would not affect the second-level statistical inference for FC, which is corrected by FDR. Our results corrected for multiple comparisons exhibited then a FC between the left hippocampus and areas of the left MTG significantly decreased in ε4(+) relative to ε4(−). This partially supports outcomes from previous research, wherein findings in FC are also mixed in the literature as it occurs with those coming from the structural analysis. For example, decreased FC from the hippocampus to the cingulate and precuneus and increased FC from the hippocampus to the sensorimotor cortex have been reported in ε4(+) contrasted with ε4(−) in cognitively healthy young adults with ages similar to those of our cohort [22]. Additionally, the same cohort studied here showed decreased FC in the DMN areas using a different methodology, specifically in the right angular gyrus and superior occipital regions in ε4(+) compared to ε4(−) [47]. By contrast, other studies undertaken with young adults aged 20 years have shown significantly increased FC in ε4(+) along trajectories that begin at the hippocampus and reach neighboring areas [48] or regions within the DMN [49].

Later in life, rsfMRI investigations have also presented mixed results. In one study, no differences in FC have been found between ε4(+) and ε4(−) aged between 20 and 40 years [50]. However, other studies that contrasted these groups reported that ε4(+) aged roughly 45 years can exhibit decreased FC between the posterior face of the hippocampi, prefrontal cortex, cingulate, precuneus, visual cortex, and lingual gyrus, whereas the anterior face of the hippocampi can present increased FC with the frontal and parietal regions [30]. Apart from this, middle-age ε4(+) from 40 to 60 years old have shown a significantly reduced global and local efficiency of the integrated resting state connectivity compared to ε4(−) [51]. Altogether, the present results are in line with those from studies conducted with cognitively healthy controls (with and without beta-amyloid pathology, which is one of the hallmarks of AD) [52], MCI patients positive for beta-amyloid pathology [52], and AD patients [53,54], where a decreased FC widespread across the MTL (especially from the hippocampal, entorhinal, and perirhinal areas) towards other multiple brain areas has been observed.

Explaining the outcomes obtained here is highly complex given the nature of the variables studied; however, some considerations can be suggested. The APOE ε4 genotype is the most important genetic risk factor for AD and may have a detrimental effect on hippocampi sizes across the lifespan that starts around the age of 20 [40]. This pattern probably leads to the decreased hippocampal volumes seen in ε4(+) in later life, whether they are cognitively healthy individuals [55], MCI patients [56,57], or AD patients [57,58]. Yet, our findings showcase a tendency in which the left hippocampus may be slightly altered in young ε4(−) contrasted with ε4(+), in accordance with the uncorrected outcomes yielded by VBM.

On the other hand, our results suggest that the left hippocampal function seems to be significantly affected in ε4(+) compared to ε4(−), which is specifically reflected by the significantly decreased FC found between the left hippocampus and the left MTG. This finding aligns with outcomes from existing studies on ε4(+) with preserved cognition, either young [59] or older [52] adults, as well as patients with MCI [52] or AD [54], which suggested similar reductions of FC between the hippocampus and the MTL. A possible explanation for the reduction of left hippocampal FC with the left MTG seen here in ε4(+) compared to ε4(−) may be associated with the specific mechanisms of the ε4 allele involved in beta-amyloid plaques and tau tangles formation [60,61]. It is presumed that this genetic predisposition to such pathologies may introduce a subtle vulnerability even in early life that could affect functional neural connections.

It may be theorized that the uncorrected increase in GM concentrations of the left hippocampus observed here in young ε4(+) is a compensatory mechanism to the decreased FC seen between the left hippocampus and the left MTG. It may be possible that young ε4(+) implement this strategy to maintain an appropriate cognitive performance. It should be noted that the described theory differs from the compensation hypothesis formulated for cognitively intact ε4(+) [62] in which an increased hippocampal activity or the engagement of other contiguous brain regions act as a neurocognitive strategy to counterbalance the potential underpinning pathology maintaining then an adequate cognitive efficiency, particularly when performing episodic memory tasks [63,64]. Our results can also be related to the ‘antagonistic pleiotropy’ theory, which postulates that certain genes or alleles “may impact fitness (i.e., survival and reproduction) differently during different life stages” [65]. If APOE ε4 was an allele with antagonistic pleiotropy, this could elucidate not only the occurrence of changes in the cognitive performances of ε4(+) over the course of a lifetime [62] but also the variability of the outcomes found in the brain structure [40] and function [66] across distinct age groups, including the young ones analyzed in this investigation.

The main limitation of this study concerns the imbalanced group size of ε4(+), which is considerably smaller than that of ε4(−), although such numbers probably represent the natural distribution of this genotype. This difference in the group sizes may account for the weak statistical power encountered here in the VBM analysis. More balanced sample sizes for both groups would have been ideal to increase the overall power. To sum up, our results must be cautiously interpreted due to their elevated complexity and the potential impact of other modifiable and non-modifiable risk factors that have not been considered in the current study. Nonetheless, it should be noticed that, to the best of our knowledge, the current cohort made up of ε4(+) and ε4(−) is still one of largest cohorts in this age range studied to date. Future studies should ideally reproduce the methodological aspects of this investigation across a much larger sample with individual APOE alleles (i.e., ε2, ε3, and ε4), which was not possible to implement in our sample. Similarly, looking into the gene-gene interaction effects of APOE ε4 and other genetic risk factors for AD on the brain structure and function can also set other interesting lines of research. Lastly, an extensive examination of FC in networks beyond the hippocampus, such as other critical regions in the medial temporal lobe network [67], can be of high interest.

## Figures and Tables

**Table 1:** Demographic data of the sample.

Parameter	Descriptive Statistics per Group				Comparisons
	Structural Neuroimaging Analysis (VBM)		Functional Neuroimaging Analysis (SBC)		
	ε4(+) (*n* = 28)	ε4(−) (*n* = 123)	ε4(+) (*n* = 27)	ε4(−) (*n* = 102)	
Age ^a^	19.53 ± 0.98	19.64 ± 0.87	19.57 ± 0.98	19.64 ± 0.87	nsd for VBM and SBC
Years of Education ^a^	12.94 ± 0.54	13.11 ± 0.57	12.95 ± 0.55	13.14 ± 0.59	nsd for VBM and SBC
Sex ^b^					nsd for VBM and SBC
%Female (n)	57.14% (16)	56.10% (69)	57.14% (16)	56.10% (69)	
%Male (n)	42.86% (12)	43.90% (54)	42.86% (12)	43.90% (54)	

Results are expressed as mean ± standard deviation. ^a^ Comparisons made with Mann–Whitney *U* tests. ^b^ Comparison made with the chi-square (X^2^) test. VBM = voxel-based morphometry; SBC = seed-based connectivity; ε4(+) = APOE ε4 carriers; ε4(−) = APOE ε4 non-carriers; nsd = non-significant differences (*p* > 0.05).

**Table 2:** Structural differences between ε4(+) and ε4(−): VBM analysis.

VBM Analysis Contrast: APOE ε4 Carriers > APOE ε4 Non-Carriers p_uncorr_ < 0.001, 97.472 Expected Voxels per Cluster											
Structure (Hemisphere)	%	Types of Significance							MNI Coordinates		
		Cluster-Level			Peak-Level						
		p_FWEcorr_	k_e_	p_uncorr_	p_FWEcorr_	T	Z_e_	p_uncorr_	mm	mm	mm
Hippocampus (Left)	50.9	0.153	534	0.023	0.493	4.00	3.89	0.000	−24	−14	−21
Posterior Insula (Left)	26.9	0.763	148	0.202	0.943	3.51	3.43	0.000	−39	−12	12

p_FWEcorr_ = family-wise error-corrected *p*-value; k_e_ = expected voxels per cluster; p_uncorr_ = uncorrected *p*-value, T = *t*-score; Z_e_ = expected Z-score.

**Table 3:** Differences in FC between ε4(+) and ε4(−): SBC analysis.

SBC Analysis between the Hippocampus Left and Other Voxels across the Brain and the Posterior Insula Left and Other Voxels across the Brain				
Seed	Associated Brain Areas	T_(123)_	p_uncorr_	p_FDR_
Hippocampus (Left)		4.59	0.000011	0.000011
	Middle Temporal Gyrus, posterior region (Left)			
	Middle Temporal Gyrus, temporooccipital division (Left)			
Posterior Insula (Left)	None	na	na	>0.05

p_uncorr_ = uncorrected *p*-value; p_FDR_ = False Discovery Rate *p*-value; T = *t*-score; Z_e_ = expected Z-score.

## Data Availability

The data supporting the findings of this study are available on request from the corresponding authors. The data are not publicly available due to privacy or ethical restrictions.
